# Restoring pars intermedia dopamine concentrations and tyrosine hydroxylase expression levels with pergolide: evidence from horses with pituitary pars intermedia dysfunction

**DOI:** 10.1186/s12917-020-02565-3

**Published:** 2020-09-25

**Authors:** Jessica S. Fortin, Matthew J. Benskey, Keith J. Lookingland, Jon S. Patterson, Erin B. Howey, John L. Goudreau, Harold C. Schott

**Affiliations:** 1 Department of Pathobiology and Diagnostic Investigation, College of Veterinary Medicine, Michigan State University, 784 Wilson Road, East Lansing, 48824 MI USA; 2 Department of Pharmacology and Toxicology, Neuroscience Program, College of Veterinary Medicine, Michigan State University, East Lansing, MI USA; 3 Neurology and Ophthalmology, College of Osteopathic Medicine, Michigan State University, East Lansing, MI USA; 4 Department of Large Animal Clinical Sciences, College of Veterinary Medicine, Michigan State University, 784 Wilson Road, East Lansing, MI 48824 USA

**Keywords:** Dopamine agonist, Equine, Parkinson disease animal model, Pituitary pars intermedia adenoma

## Abstract

**Background:**

Pituitary pars intermedia dysfunction (PPID) develops slowly in aged horses as degeneration of hypothalamic dopaminergic neurons leads to proliferation of pars intermedia (PI) melanotropes through hyperplasia and adenoma formation. Dopamine (DA) concentrations and tyrosine hydroxylase (TH) immunoreactivity are markedly reduced in PI tissue of PPID-affected equids and treatment with the DA receptor agonist pergolide results in notable clinical improvement. Thus, we hypothesized that pergolide treatment of PPID-affected horses would result in greater DA and TH levels in PI tissue collected from PPID-affected horses versus untreated PPID-affected horses.

To test this hypothesis, pituitary glands were removed from 18 horses: four untreated PPID-affected horses, four aged and four young horses without signs of PPID, and six PPID-affected horses that had been treated with pergolide at 2 µg/kg orally once daily for 6 months. DA concentrations and TH expression levels in PI tissues were determined by high performance liquid chromatography with electrochemical detection and Western blot analyses, respectively.

**Results:**

DA and TH levels were lowest in PI collected from untreated PPID-affected horses while levels in the pergolide treated horses were similar to those of aged horses without signs of PPID.

**Conclusions:**

These findings provide evidence of restoration of DA and TH levels following treatment with pergolide. Equine PPID is a potential animal model of dopaminergic neurodegeneration, which could provide insight into human neurodegenerative diseases.

## Background

Pituitary Pars Intermedia Dysfunction (PPID), a naturally occurring, slowly progressive neurodegenerative disorder affecting older horses and ponies, is the most commonly diagnosed equine endocrinopathy [1–4]. The hallmark clinical sign of PPID is hypertrichosis; an abnormally long, shaggy and often curly coat that fails to shed [5]. Additional signs associated with PPID include muscle wasting, abnormal fat distribution, lethargy, infertility, and an increased susceptibility to both parasitic and bacterial infections, which can lead to sinusitis, dermatitis, endoparasitism and laminitis [3, 4, 6–9]. The latter, laminitis, is a painful disorder of the feet that often necessitates euthanasia [4]. Several studies have also found that insulin dysregulation may accompany some cases of PPID, leading to glucose intolerance, type II diabetes, polydipsia and polyuria. Insulin dysregulation is also thought to predispose PPID-affected horses to laminitis [10]. Furthermore, up to a third of PPID-affected horses have been observed to display neurological deficits, including obtundation and seizure-like activity. Post-mortem examination of PPID afflicted horses reveals enlargement of the pituitary gland (PG) due to hyperplasia, hypertrophy and adenoma formation in the pars intermedia (PI). Some studies have reported up to a three-fold increase in PG weight in horses with PPID [1, 9].

The PI is composed of a single cell type known as a melanotrope, which produces pro-opiomelanocortin (POMC). Within melanotropes, POMC is differentially cleaved into the main secretory products of the PI; α-melanocyte-stimulating hormone (α-MSH), β-endorphin and corticotrophin-like intermediate lobe peptide (CLIP). In addition, a small amount of POMC may also be cleaved into adrenocorticotropin (ACTH); however, the majority of circulating ACTH is produced by the pars distalis of the PG [1, 10–12]. In horses with PPID, hyperplasia, hypertrophy and adenoma formation of PI melanotropes results in a large increase in POMC synthesis with corresponding increases in release of α-MSH, β-endorphin, CLIP, and a comparatively smaller increase in ACTH release [10]. Although the mechanism(s) by which these POMC-derived peptides lead to development of clinical signs is not well understood, it may involve interactions between α-MSH, β-endorphin and ACTH. For example, α-MSH and β-endorphin are capable of inducing a six-fold increase in the steroidogenic properties of ACTH [13]. Thus, a small increase in ACTH coupled with larger increases in potentiating peptides, α-MSH and β-endorphin, may be sufficient to stimulate adrenocortical steroidogenesis, resulting in increased plasma cortisol levels and loss of circadian rhythmicity of cortisol secretion [10, 14]. Hence, horses with PPID are commonly referred to as having equine Cushing’s disease. However, Cushing’s disease parameters do not incorporate all the features of PPID and thus does not adequately explain equine PPID.

The exact cause of PPID is unknown, however, loss of dopaminergic innervation to the PI seems to be critical in development of the disorder. Normally, dopaminergic innervation from the periventricular nucleus (PVN) of the hypothalamus inhibits POMC expression in the PI via activation of dopamine (DA) D_2_ receptors on melanotropes [11, 12, 15, 16]. In horses with PPID, progressive loss of dopaminergic inhibition results in an increase in circulating POMC derivatives. Although the mechanisms for loss of dopaminergic innervation remain uncertain, oxidative stress and mitochondrial dysfunction in PI have been suggested to play a role [1, 17]. Concentrations of DA and DA metabolites have been found to be 9-fold lower in PI tissue of horses with PPID as compared with normal horses [18]. In addition, loss of hypothalamic (periventricular nuclei, PVN) dopaminergic neurons has been found in PPID-affected horses, as compared to aged horses without PPID [1]. Further, DA D_2_ receptor knockout mice develop PI hypertrophy and hyperplasia similar to the histopathologic changes in the PG found with PPID in horses [19].

In horses with PPID, PI tissue has further been shown to have increased amounts of immunochemical staining for 3-nitrotyrosine [1]. In human medicine, accumulation of 3-nitrotyrosine occurs in neurodegenerative diseases such as Parkinson’s disease [20–22], but the significance is equivocal since similar findings have been described in healthy aged individuals [23]. In familial Parkinson’s disease, a mutation in the gene encoding α-synuclein has been correlated with cytoplasmic accumulation of α-synuclein in Lewy Bodies that is thought to contribute to degeneration of nigrostriatal dopaminergic neurons [24–26]. Interestingly, nitrated α-synuclein has been shown to be more neurotoxic than native α-synuclein [27]. Horses with PPID have higher levels of nitrated α-synuclein in the dopaminergic nerve terminals within the PI compared with healthy horses [1]. Decreased TH immunoreactivity has also been described in the PI of PPID-affected horses [1, 17, 18]. Loss of TH immunoreactivity also occurs in Parkinson’s disease, although the role of this finding in the pathophysiology of Parkinson’s disease is not defined [28]. Equine PPID shares similar neurochemical perturbations observed in Parkinson’s disease including oxidative stress, antioxidant deficiency, nitrated α-synuclein accumulation and loss of TH immunoreactivity. Thus, equine PPID may have potential as an animal model to better understand the neurobiology of dopaminergic neurodegenerative diseases, including Parkinson’s disease.

In humans, pharmacotherapy of Parkinson’s disease is primarily aimed at restoring dopaminergic neurotransmission in the corpus striatum. Currently, the most effective treatment is levodopa in combination with a peripheral decarboxylase inhibitor [29]. Levodopa is metabolized into DA in dopaminergic neurons, temporarily restoring levels of this endogenous neurotransmitter. Long-term use of levodopa is associated with fluctuation of motor responses and dyskinesias [30, 31], thus narrowing the therapeutic window [32]. Levodopa is also known to be neurotoxic *in vitro* [33]. *In vivo* studies demonstrate formation of cytotoxic free radicals with decarboxylation of levodopa. As such, increasing levels of DA through administration of levodopa could potentially cause oxidative damage to surviving dopaminergic neurons, creating a risk of eventually exacerbating the disease [34, 35]. DA agonists were introduced to mimic the endogenous neurotransmitter [36], and as an adjunct to levodopa treatment in patients exhibiting fluctuating motor responses and dyskinesias due to chronic use of levodopa [37–39]. DA agonist administration reduces the dose of levodopa required by 20%-30% as well as the disabling complications. There are two subclasses of DA agonists targeting DA D_2_-type receptors: ergoline agonists (pergolide, bromocriptine, lisuride, and cabergoline) and non-ergoline agonists (ropinirole and pramipexole). DA agonists are not metabolized by oxidative pathways and do not generate cytotoxic free radicals associated with metabolism of DA or levodopa. These drugs may further protect dopaminergic neurons from injury resulting from levodopa by suppressing endogenous DA release. DA agonists cause less motor complications versus levodopa, possibly due to a longer half-life and differences in receptor selectivity [40].

Pergolide has been shown to improve symptoms of Parkinson’s disease alone or in combination with levodopa and may be more effective than bromocriptine [41–43]. This could be explained by the action of pergolide on both DA D_1_ and DA D_2_-type receptors, whereas bromocriptine is a DA D_2_ agonist with weak DA D_1_ antagonistic properties [41]. In veterinary medicine, administration of pergolide is commonly used as a therapeutic agent in horses afflicted with PPID. Pergolide treatment results in a decrease in plasma concentrations of POMC derived peptides and notable clinical improvement [44]. We hypothesized that DA concentrations and TH expression levels are greater in PI tissue collected from PPID-affected horses treated with pergolide than those in PI tissues collected from untreated PPID-affected horses. PPID causes depletion of DA and treatment with a DA agonist would allow the dopaminergic neurons to replenish DA storage in vesicles. In rodents, activation of DA D1 and D2 receptors has been shown to contribute to increasing TH enzymatic activity [45]. Thus, in the current study, DA concentrations and TH expression levels in PI tissue were compared between PPID-affected horses treated with pergolide over a period of 6 months, untreated PPID-affected horses, untreated aged horses without clinical signs of PPID, and untreated young horses. Our results suggest that equine PPID is a potential animal model for DA neurodegeneration which could give insights to Parkinson’s disease and provides additional evidence of the therapeutic benefits of dopaminergic receptor agonists.

## Results

**Fig. 1:**
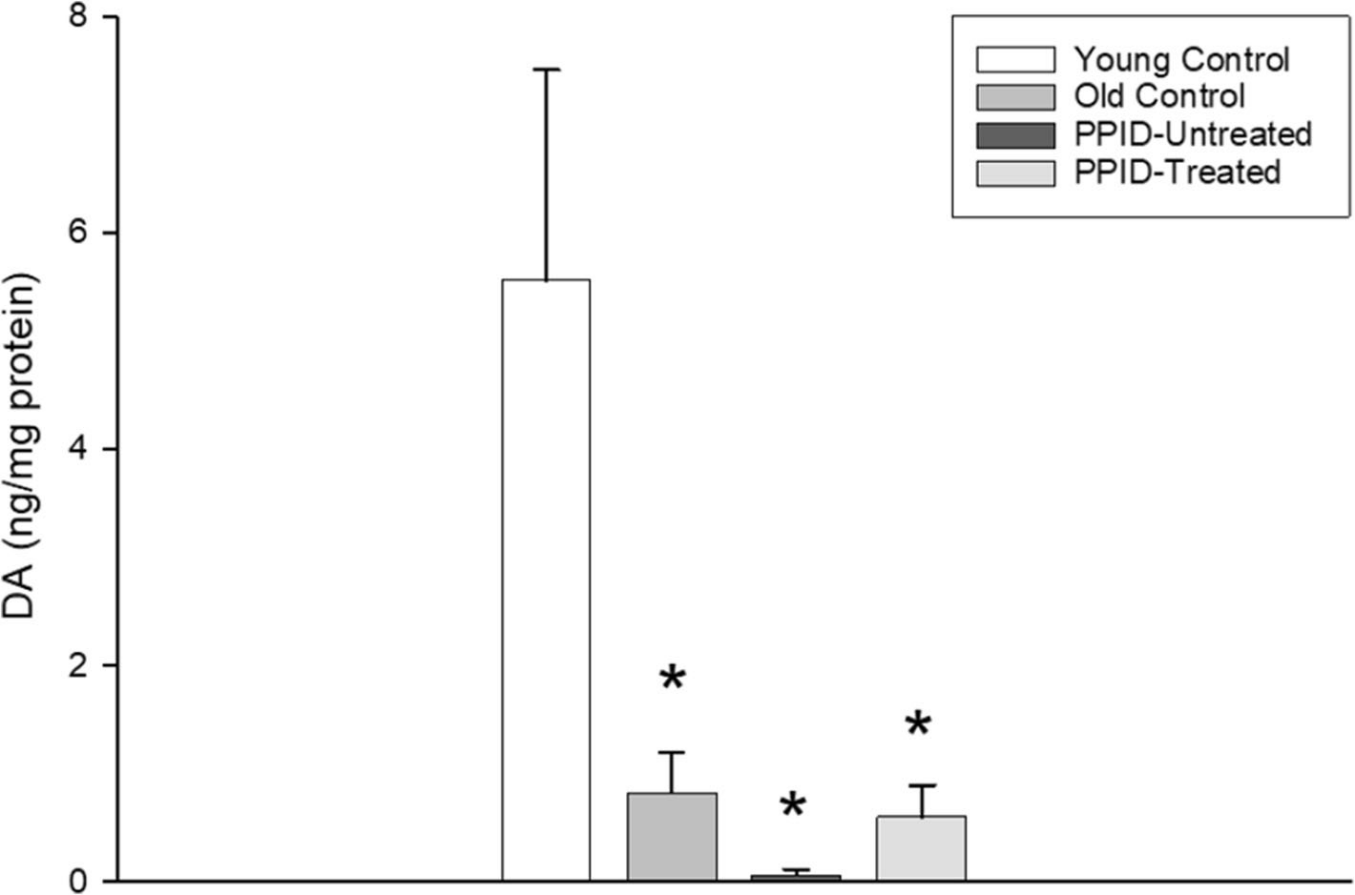
A 6-month treatment with pergolide restores the concentration of DA in the pars intermedia of PPID horses to the level of comparably aged horses without PPID. DA concentrations were measured in the pars intermedia of pituitary glands using HPLC-ED. Pituitary glands were collected from horse controls (young and old normal control horses), PPID untreated horses, and PPID afflicted horses that had received treatment with pergolide (DA agonist) at 2 µg/kg per os for 6 months. Columns represent mean DA concentrations + S.E.M. * Values that are significantly different from the young control group (***p ***< 0.05)

This study focused on measuring DA concentrations in PI tissue collected from horses of different age and disease status to understand the impact of DA agonist treatment in PPID-affected horses. A summary of groups with the associated PG weights and histologic score are presented in Table 1. All 10 PPID-affected horses had hypertrichosis and other clinical signs of PPID. Treatment with pergolide did not reduce the weight of the PGs (Table 1). DA concentrations were higher for the healthy young horses (5.6 ± 1.9 ng/mg) compared with all other groups, with mean DA concentration in PI tissue collected from all aged horses about 5 times less than that of young horses (Fig. 1).

**Table 1:** Mean horse age and pituitary gland weights and histologic grades for each group

Groups of horses (n)	Age Mean (year) ± SD	Weight Mean (g) ± SD	Histologic grade Median ± Range (# adenoma/n)
**Young (4)**	5 ± 2	2.21 ± 0.19	2 ± 1 (0/4)
**Old (4)**	25 ± 5	3.14 ± 1.30	4 ± 2 (1/4)
**Untreated PPID (4)**	27 ± 4	6.89 ± 2.70	5 ± 0 (4/4)
**Treated PPID (6)**	26 ± 3	5.28 ± 1.10	5 ± 0 (6/6)

Mean DA concentration was lowest in the PPID untreated group (0.06 ± 0.06 ng/mg) (Fig. 1). Interestingly, there was no difference between PI DA concentrations in pergolide treated PPID horses (0.60 ± 0.29 ng/mg) as compared with similar aged horses without clinical signs of PPID (0.82 ± 0.37 ng/mg), suggesting treatment with pergolide restored DA concentrations in the PI to age-appropriate levels.

**Fig. 2:**
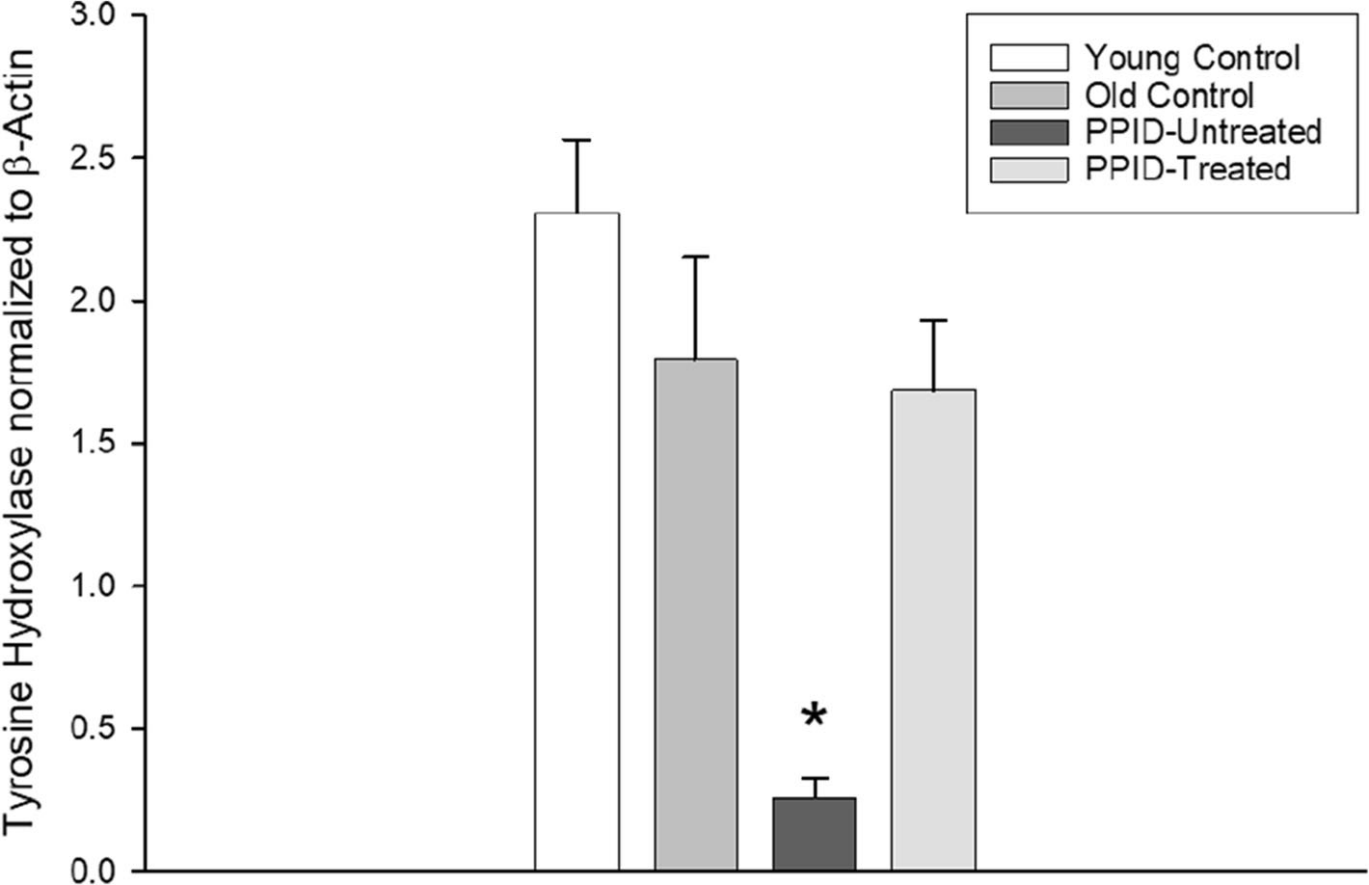
TH expression levels are re-established in the pars intermedia of PPID horses on a 6-month pergolide regimen. TH expression levels were determined in the pars intermedia of pituitary glands using western blot. Pituitary glands were collected from horse controls (young and old normal control horses), PPID untreated horses, and PPID afflicted horses that had received treatment with pergolide (DA agonist) at 2 µg/kg per os for 6 months. Columns represent mean TH concentrations normalized to β-actin + S.E.M. * The PPID-untreated value is significantly different from the young and old control groups as well as the PPID-treated group (***p*** < 0.05)

In this study, we investigated if there is loss of TH expressions levels with neurodegeneration of hypothalamic PVN dopaminergic neurons that innervate the PI. TH expression levels were decreased in untreated-PPID horses (Fig. 2). However, there was no significant difference in TH concentration in PI tissue between the young and aged horses without PPID. PPID horses treated with pergolide showed similar PI TH expression levels as young and aged horses without PPID (Fig. 2). These results suggest that pergolide treatment re-establishes TH expression levels in PI of PPID-affected horses.

## Discussion

We hypothesized that pergolide treatment would re-establish DA and TH concentrations in PI tissue in horses with PPID. Our data are consistent with this hypothesis; we demonstrated that horses with PPID that received pergolide treatment had partial (compared with young horses) and complete (compared with aged horses) recovery of DA concentration in the PI. However, this study would need to be repeated with higher numbers of horses per group to confirm the absence of significant difference in DA concentration between PPID and non PPID horses. In addition, pergolide treated horses showed complete recovery of TH protein concentrations in the PI. Loss of hypothalamic dopaminergic innervation has a role in PPID physiopathology that has not yet been fully defined. Administration of DA agonists is a logical therapeutic strategy for horses with PPID [46–48], which our data further reinforce. Previous work has shown that PPID horses treated with DA agonists have decreased levels of POMC peptides and adrenocorticotropic hormone (ACTH) in the PI, and cortisol concentrations in plasma [14, 46, 49].

Our study focused on the determination of DA concentration in the PI because the PG receives direct innervation from the dopaminergic neurons of the PVN of the hypothalamus. These axons project through the infundibular stalk, travel along the periphery of the nerve pathway and then terminate in the PI. At this site, DA, released by the periventricular nerve terminals, interacts with melanotrope DA D2 receptors causing a decrease in POMC-derived hormone synthesis and release, as well as inhibition of cell division [50]. There is mounting evidence that PPID is a neurodegenerative disorder characterized by loss of DA neurons and, subsequently, dopaminergic input to PI melanotropes. The lack of endogenous DA-mediated inhibition of PI POMC production in PPID-affected equids may also result in multiclonal expansion which could explain (in addition to the fluctuation of cortisol release in the blood stream [14]) PI hyperplasia and formation of multifocal adenomas via persistent hormonal stimulation. Dorsal expansion of the PG and compression of surrounding structures including the hypothalamus and infundibular stalk might explain, in part, loss of PVN dopaminergic axon terminals which decreases the concentration of DA and DA-metabolites [1]. In the present study, untreated PPID-affected horses had **~** 10-fold less DA in PI tissue as measured by HPLC-ED, which provides biochemical confirmation of the immunohistochemical data published by McFarlane et al. [1].

Our study provides additional evidence that PPID-affected horses have significantly decreased PI DA concentrations. Interestingly, aged horses without PPID have reduced DA concentrations in comparison to young horses. A previous study identified reduced numbers of dopaminergic cell bodies in the hypothalamic PVN of PPID-affected horses compared to healthy aged horses [1]. Aging decreases DA concentrations in the spinal cord of adult mares compared to pre-pubertal females [51]. In the present study, PI DA concentrations of PPID-affected horses treated with pergolide were not restored to the same level as young horses. This may be due to normal age-related loss of dopaminergic neurons, as supported by enlargement of the PI in aged horses without clinical signs of PPID. It would have been useful to include a group of aged horses without PPID that received pergolide treatment to provide further evidence of DA restoration in the context of age-related DA-neurodegeneration.

In a previous study, TH immunoreactivity was reduced in PI tissues obtained from PPID horses and no difference was detected in TH between young and aged horses using immunohistochemistry techniques [1]. This finding is consistent with a role for dopaminergic neurodegeneration in the etiology of PPID and prompted evaluation of TH expression levels in PI tissues from different horse groups included in this study. Similarly, TH protein expression level by Western blot analyses in the current study was similar in young and aged horses. Mechanisms regulating TH expression and DA synthesis are distinctly different, which might explain the difference observed between and TH concentrations (no change) and DA concentrations (decreased) in the aged horses without PPID. TH is an enzyme that catalyzes the first and rate-limiting step in biosynthesis of catecholamines (such as DA), converting tyrosine to L-dihydroxyphenylalanine (L-DOPA). TH is localized in presynaptic catecholaminergic neurons in general, including noradrenergic neurons, and not solely expressed in dopaminergic neurons [52]. TH activity is controlled by direct regulation (short term regulation) of enzyme activity (allosteric regulation, catecholamine feedback inhibition and phosphorylation) and gene transcription (long term regulation) [52]. In the present study, TH expression levels in aged horses remained similar to those of young horses in contrast to lower DA concentrations in aged horses. This finding highlights the independent synthetic pathways, turn over and genetic expression of DA receptors and TH in equid PI. Activation of DA D1 and D2 receptors in rodents has been shown to increase TH S-nitrosylation and its enzymatic activity [45]. It would be interesting in a further study to assess TH enzymatic activity and posttranslational modifications such as phosphorylation and nitrosylation in PI of young, old, and PPID-affected horses to determine if one of these modifications could potentially result in a difference in TH activity. This might explain the low concentration of DA, despite normal TH expression levels in aged horses.

In the current study, a 6-month oral treatment with pergolide re-established the concentrations of DA and TH in PI tissue of PPID-affected horses to levels equivalent with aged horses without clinical signs of PPID. In Parkinson’s disease, dysfunction of the nigrostriatal tract begins with an initial loss of TH expressing nigrostriatal axon terminals and is followed by actual loss of dopaminergic neurons in the substantia nigra [53, 54]. Altered DA synthesis in the striatum causes loss of fine motor skills [53]. Novel therapeutic interventions in Parkinson’s disease focus on halting or reversing neurodegeneration of the nigrostriatal DA neuronal tract with gene therapy. Aromatic l-amino acid decarboxylase (AADC) gene therapy with an adeno-associated viral vector that can convert peripheral l-dopa to dopamine has been shown to restore DA production in Parkinsonian monkeys [55]. Previous work in the 6-hydroxydopamine rat model of experimental Parkinson’s disease demonstrated complete normalization of striatal TH activity following an intravenous administration of transferrin receptor monoclonal antibody (TfRmAb) targeted pegylated immunoliposomes carrying a TH expression plasmid [56]. Our data provide initial evidence that a DA agonist could actually re-establish TH protein expression in dopaminergic neurons. The specific mechanism for increases in DA concentration and TH expression level following pergolide treatment remains unclear. One explanation could be that PPID causes depletion of DA and treatment with a DA agonist allows dopaminergic neurons to replenish DA storage in vesicles. Activation of DA D1 and D2 receptors may lead to increase of TH enzymatic activity. Our data further suggest that similar pharmacological strategies could be employed to restore DA biosynthesis in Parkinson’s disease.

## Conclusions

The results of this study demonstrate that pergolide, a DA agonist, re-establishes DA and TH levels in the PI of PPID-affected horses. The ability of pergolide to re-establish a dopaminergic phenotype of aged horses without PPID provides support for further investigation of the therapeutic efficacy of dopaminergic receptor agonists for other dopaminergic neurodegenerative disorders, including Parkinson’s disease.

## Methods

### Animals

Horses were owned by another individual/farm and informed written consent was obtained from the owners. All procedures were approved by the Michigan State University Institutional Animal Care and Use Committee (AUF 11/08-191-00). Horses were sedated with xylazine HCl (0.6 mg/kg, IV) and Na pentobarbital (100 mg/kg, IV) was administrated for euthanasia. Pituitary glands were removed within 30 min of euthanasia from 18 horses in the fall (August to November): six PPID-affected horses (26 ± 3 years) that were treated with pergolide at 2 µg/kg per os once daily for 6 months, four untreated PPID-affected horses (27 ± 4 [SD] years), four aged (25 ± 5 years) horses without signs of PPID, and four young (5 ± 2 years) normal horses.

### PPID status

PPID status was confirmed by presence of clinical signs, notably hypertrichosis. Postmortem histologic examination of the PG was performed by two veterinary pathologists using the histologic grades established by Miller et al. as follows: (1) normal; (2) focal to multifocal PI hypertrophy or hyperplasia; (3) diffuse PI adenomatous hyperplasia; (4) PI adenomatous hyperplasia with microadenomas 1–5 mm in diameter; (5) adenoma > 5 mm in the PI [3].

### Tissue preparation

Frozen PGs (-80°C) were examined to determine rostral-caudal orientation and sagittal sections (500 µM thick) were sliced from each PG starting from an abaxial border. Central sections were viewed under a dissecting microscope in which the three separate lobes of the PG (pars distalis, PI and pars nervosa) could be discerned. Samples (two 18 gauge needle punches) were obtained from the PI and prepared for neurochemical analyses. Samples were placed into microtubes containing 65 µL of ice cold 0.1M phosphate-citrate buffer containing 15% methanol. Tissues were sonicated with three short (≈ 1 sec) bursts to release monoamines stored within vesicles. Samples were next centrifuged for 1 minute. Supernatants (containing monoamines) were extracted with a 100 µl Hamilton syringe, placed in a new microtube, and centrifuged for 5–10 sec. A volume of 100 µL 0.1 M NaOH was then pipetted into the microtube containing pellet (proteins). Supernatants were stored at -20 °C until HPLC-ED analysis and pellets were stored at room temperature until Lowry protein assay was conducted.

### Dopamine (DA) concentration determination by high performance liquid chromatography with electrochemical detection (HPLC-ED)

For measurement of DA using HPLC-ED, 10 µL of each supernatant were injected into C-18 columns. The HPLC mobile phase was a 20% methanol and 0.02% SOS solution with a pH of 2.65. PI samples were compared to a 1 ng “7-mix” standard containing dihydroxyphenylethylene glycol (DOPEG), norepinephrine (NE), 3,4-dihydroxyphenylacetic acid (DOPAC), homovallinic acid (HVA), DA, serotonin (5HT) and 5-hydroxyindoleacetic acid (5HIAA). PI DA concentrations were expressed in ng/mg protein, using protein content values obtained from a Lowry protein assay.

### Tyrosine-hydroxylase (TH) expression levels by western blot analysis

PI protein extracts (15 µg) were solubilized in SDS-PAGE buffer (62.5 mM Tris pH 6.8, 2% SDS, 10% glycerol, 0.00125% bromophenol blue, and 15% β-mercaptoethanol). The extracts were boiled for 5 min, separated (15 µg) on 10% SDS-PAGE electrophoresis gel and transferred onto a nitrocellulose membrane. All membranes were blocked and incubated with an appropriate dilution of the primary antibody: TH antibody (1:2000; Millipore AB152) and β-actin (1:1000; Cell Signaling #3700). Membranes were next washed and incubated with a horseradish peroxidase-conjugated goat anti-mouse or anti-rabbit IgG secondary antibody (1: 2500; (Cell Signaling) followed by chemiluminescent detection, using an enhanced chemiluminescence (ECL) detection kit (ThermoFisher Scientific). Films were scanned and the density of each band was measured with Image J software. TH expression levels were normalized to β-actin.

### Statistical analysis

Factorial-ANOVA statistical comparisons between groups were made using Sigma Stat software version 2.03 (SysStat Software, Inc.). A *p* value of less than or equal to 0.05 was considered significant. If a statistical difference was detected, *post hoc* analysis was performed using Tukey’s Test for between group comparisons.

## Data Availability

The data used and/or analyzed in the present study are available from the corresponding author on reasonable request.

## Abbreviations

5HIAA: 5-hydroxyindoleacetic acid
5HT: Serotonin
α-MSH: α-melanocyte-stimulating hormone
AADC: Aromatic l-amino acid decarboxylase
ACTH: Adrenocorticotropic hormone
CLIP: Corticotrophin-like intermediate lobe peptide
DA: Dopamine
DOPAC: 3,4-dihydroxyphenylacetic acid
DOPEG: Dihydroxyphenylethylene glycol
ECL: Enhanced chemiluminescence
HPLC-ED: High performance liquid chromatography with electrochemical detection
HVA: Homovallinic acid
L-DOPA: L-dihydroxyphenylalanine
NE: Norepinephrine
ODST: Overnight dexamethasone suppression test
PI: Pars intermedia
PG: Pituitary gland
POMC: Pro-opiomelanocortin
PPID: Pituitary pars intermedia dysfunction
PVN: Periventricular nucleus
TfRmAb: Transferrin receptor monoclonal antibody
TH: Thyrosine hydroxylase
